# Dorsolateral prefrontal cortex dysfunction caused by a go/no-go task in children with attention-deficit hyperactivity disorder: A functional near-infrared spectroscopy study

**DOI:** 10.3389/fnins.2023.1145485

**Published:** 2023-03-28

**Authors:** Ting Wu, Xiaoli Liu, Fang Cheng, Shuai Wang, Chang Li, Dongsheng Zhou, Wenwu Zhang

**Affiliations:** Affiliated Mental Health Centre & Ningbo Kangning Hospital, Ningbo University, Ningbo, Zhejiang, China

**Keywords:** functional near-infrared spectroscopy (fNIRS), attention deficit hyperactivity disorder (ADHD), go/no-go task, dorsolateral prefrontal cortex (DLPFC), children

## Abstract

**Background:**

Children with attention-deficit hyperactivity disorder (ADHD) exhibit executive function deficits, which can be attributed to a dysfunction in the prefrontal region of the brain. Our study aims to evaluate the alteration of brain activity in children with ADHD during the administration of a go/no-go task using functional near-infrared spectroscopy (fNIRS) in comparison to a control group containing typically developing (TD) children.

**Methods:**

32 children with ADHD and 31 of their TD peers were recruited and asked to perform a go/no-go task while undergoing measurements, with the aim of detecting changes in average oxygenated hemoglobin signaling (Δavg oxy-Hb) *via* fNIRS in the prefrontal lobe.

**Results:**

fNIRS data showed significant differences between the left and right dorsolateral prefrontal cortices, with a lower Δavg oxy-Hb change in the ADHD group compared to the TD group.

**Conclusion:**

Our results indicate that brain dysfunction in children with ADHD is related to functional impairments in the dorsolateral prefrontal cortex. The go/no-go task paired with fNIRS represents a useful measurement tool to assess prefrontal brain dysfunction in children struggling with ADHD.

## Introduction

Attention-deficit hyperactivity disorder (ADHD) is one of the most common neurodevelopmental disorders in children and is characterized by age-inappropriate symptoms of inattention, impulsivity, and hyperactivity with a prevalence of 6.29% in both children and adolescents (Posner et al., 2020). Children with ADHD are often academically impaired and suffer from poor social and occupational functioning.

Several studies have been conducted to determine the cognitive basis of ADHD and have led to a plethora of theoretical descriptions for “core” deficits. However, one influential model was developed by Barkley in 1997, who assumed that response inhibition was the core deficit in children with ADHD, which in turn affected other executive functions (Barkley, 1997). Moreover, response inhibition has features consistent with several cognitive processes, such as sustained attention, rule maintenance, and target detection (Aron and Poldrack, 2005). Furthermore, ADHD is characterized by persistent inattention, which may present itself as a persistent impairment in reaching goals or the inability to maintain task orientation due to impaired self-regulation and governance. More specifically, inattention in people with ADHD may be due to poor interference control, allowing other external and internal events to override executive functions that provide self-control. This further leads to executive dysfunction in other cognitive areas such as working memory, speech internalization, emotional motivation control, and behavioral reconstruction. These deficits may affect the child's development during entry into early adulthood.

In the context of neurodevelopmental disorders, an increasing number of ADHD studies have focused on structural and functional deficits. For example, Posner and Petersen in 1990 and 2007 stated that attention consists of three independent neural networks including alertness, orientation, and executive control (Posner and Petersen, 1990; Posner and Rothbart, 2007). Alertness is the achievement and maintenance of an alert state. Orientation is defined as selective attention to target stimuli. Executive control is the effort someone puts forth to control attention (Posner et al., 2016). These networks are supported by separate regions of the brain. The brain's alertness network is associated with the frontal and parietal regions, whereas the orientation network is mainly connected to the frontal ocular field and the upper and lower parietal regions. The executive control network is controlled by the anterior cingulate gyrus and the dorsolateral prefrontal cortex (Fan et al., 2005; Rueda et al., 2015). These three networks are supposed to improve the child's task performance over the course of their childhood (Schmidt et al., 2016). Based on this theory, numerous behavioral and neuroimaging studies have been conducted on children with ADHD. Furthermore, there is growing evidence of vigilance and executive network impairments in children with ADHD, based on behavioral and neurobiological findings (Berger and Posner, 2000; Cao et al., 2008; Lambek et al., 2011; Arora et al., 2020). Despite the notable contributions of extant studies on the role of alertness in ADHD, this network is still regarded as a purely biological function, while comparatively few studies have been performed on the neural substrates responsible for deficits in executive functions in children with ADHD. Therefore, the neural mechanism of attention control in patients with ADHD remains unclear to this day.

Previous studies on the neural mechanism of executive function in children with ADHD mainly utilized behavioral science, electroencephalograms (EEGs), even correlation potentials (ERPs), and functional magnetic resonance imaging (fMRI), in addition, to other methods to compare the differences between children with ADHD and those without. (Iaboni et al., 1995; Homack and Riccio, 2004; Schachar et al., 2004; Gupta and Kar, 2009; Pievsky and McGrath, 2018). Furthermore, EEG studies have revealed that an increased resting θ/α and θ/β wave ratio in the frontal and central brain regions of children with ADHD is associated with compromised networks (Schutter et al., 2006; Lansbergen et al., 2011). Previous ERP studies have shown that children with ADHD show impaired task performance during the execution of executive function tasks along with reduced activation of their P3 components compared with patients without ADHD (Pontifex et al., 2013; Hung et al., 2016). These findings revealed several electrophysiological characteristics of executive function development in children with ADHD, which may result from abnormalities in the frontostriatal network (Emond et al., 2009; Jiang et al., 2018). Furthermore, several compelling studies combining functional magnetic resonance imaging (fMRI) and cognitive tasks revealed that impaired performance is potentially due to the decreased activation of prefrontal areas of the brain in children with ADHD compared to normal controls (Konrad et al., 2006; Cao et al., 2008; Burgess and Braver, 2010; McCarthy et al., 2014). Moreover, impaired large-scale functional connectivity has been observed in children with ADHD, indicating dysfunction in executive control-related networks (Rubia, 2018; Li et al., 2020). Previous neuroimaging studies also supported the notion of dysfunctional attention networks as the likely cause of deficient attention control in patients with ADHD, especially with regard to the right inferior frontal cortex (Aron and Poldrack, 2005). The application of these methods has allowed researchers to make progress in the study of the role of ADHD on executive function. However, due to the shortcomings of these methods, many new techniques need to be developed and employed to make further progress.

An excellent approach to studying the potential relationship between changes in brain activation and executive function in patients with ADHD is to employ functional near-infrared spectroscopy (fNIRS). fNIRS is a non-invasive neuroimaging technique that uses near-infrared light to measure changes in oxy- and deoxyhemoglobin (oxygen-Hb and deoxy-Hb) concentrations over time. It is an indirect method of measuring attention control processing throughout the performance of different neuropsychological tasks (Cui et al., 2011; Boas et al., 2014). Furthermore, fNIRS has been widely used in research due to its advantages of portability, safety, low cost, low body fixation, and freedom of patient movement.

Studies of classical paradigms such as the Stroop task, the go/no-go task, and the stop signal task have shown that response inhibition is one of many tasks that can distinguish patients with ADHD from patients with TD (Castellanos and Tannock, 2002). Two recent studies using fNIRS detected reduced activity in both the left and right DLPFC (Negoro et al., 2010; Miao et al., 2017). Furthermore, other studies reported that inhibitory tasks led to increased activity in the left DLPFC (Moser et al., 2009; Suzuki et al., 2017). Based on this evidence, PFCs must be involved in directing brain attention resources to goal-related stimuli (Miller and Cohen, 2001; Brosnan and Wiegand, 2017). Therefore, we expect changes in PFC activation in children with ADHD compared to TD control. This study aims to utilize fNIRS for the exploration of inhibitory-related hemodynamic responses in subjects performing a go/no-go task.

## Materials and methods

### Subjects

A total of 63 right-handed children between the ages of 8 and 13 were recruited for the study. The children were divided into two groups consisting of 32 children with ADHD (28 boys and four girls) and 31 TD children (24 boys and seven girls). Children with ADHD were recruited from patients referred to the Child and Adolescent Psychology Clinic of Ningbo Kangning Hospital. All children with ADHD were previously diagnosed based on the Diagnostic and Statistical Manual of Mental Disorders, Fifth Edition (DSM-5, American Psychiatric Association, 2013) by a children's developmental and behavioral pediatrician with experience in ADHD. Paid volunteers were recruited from neighboring elementary and junior high schools *via* a WeChat advertisement. In addition, the inclusion criteria for this study were Chinese ethnicity. Exclusion criteria for this study included bipolar disorder, psychosis, autism, severe obsessive-compulsive disorder, Tourette's syndrome, birth injury, head trauma, or major causative genetic, neurological, metabolic, or infectious illnesses, as well as an IQ of <80. The intelligence quotient was estimated by the Chinese version of the Wechsler Intelligence Scale for Children, Second Edition (C-WISC) (Gong and Cai, 1993). The primary measurements for assessing ADHD core symptoms were by using the Swanson, Nolan, and Pelham Rating scale (SNAP-IV), a 26-item parenting scale that included an incentive score, a 9-item hyperactivity/impulsivity score, and a 9-item oppositional score. The SNAP-IV has previously been shown to be a valid outcome measure for use in randomized controlled trials and clinical settings (Hall et al., 2020).

### fNIRS measurements

We investigated inhibition related to hemodynamic activation using a 48-channel near-infrared optical imaging system (NirScan-6000A, Danyang Huichuang Medical Equipment Co., Ltd, China) operating at three wavelengths (730/808/850 nm) with a sampling rate of 11 Hz. An elastic cap containing 15 light source optodes and 16 light detector probes was arranged, as shown in **Figure 2**, and placed on the head of each subject. The center of the middle probe was placed at electrode FPZ, while the channels corresponding to the left and right prefrontal cortexes were located along Fp1 and Fp2. The source optode and detector probe were separated by a distance of 3.0 cm. The 48 channels were divided among each brain region based on equipment coordinates, thus allowing for the selection of specified regions of interest (ROI) in this study. The regions of interest in this study were the right and left dorsolateral prefrontal cortex (DLPFC), medial prefrontal cortex (mPFC), and temporal lobe (TL). The channels corresponding to each brain region are presented in Supplementary Figure 1.

### Go/no-go task

**Figure 1:**
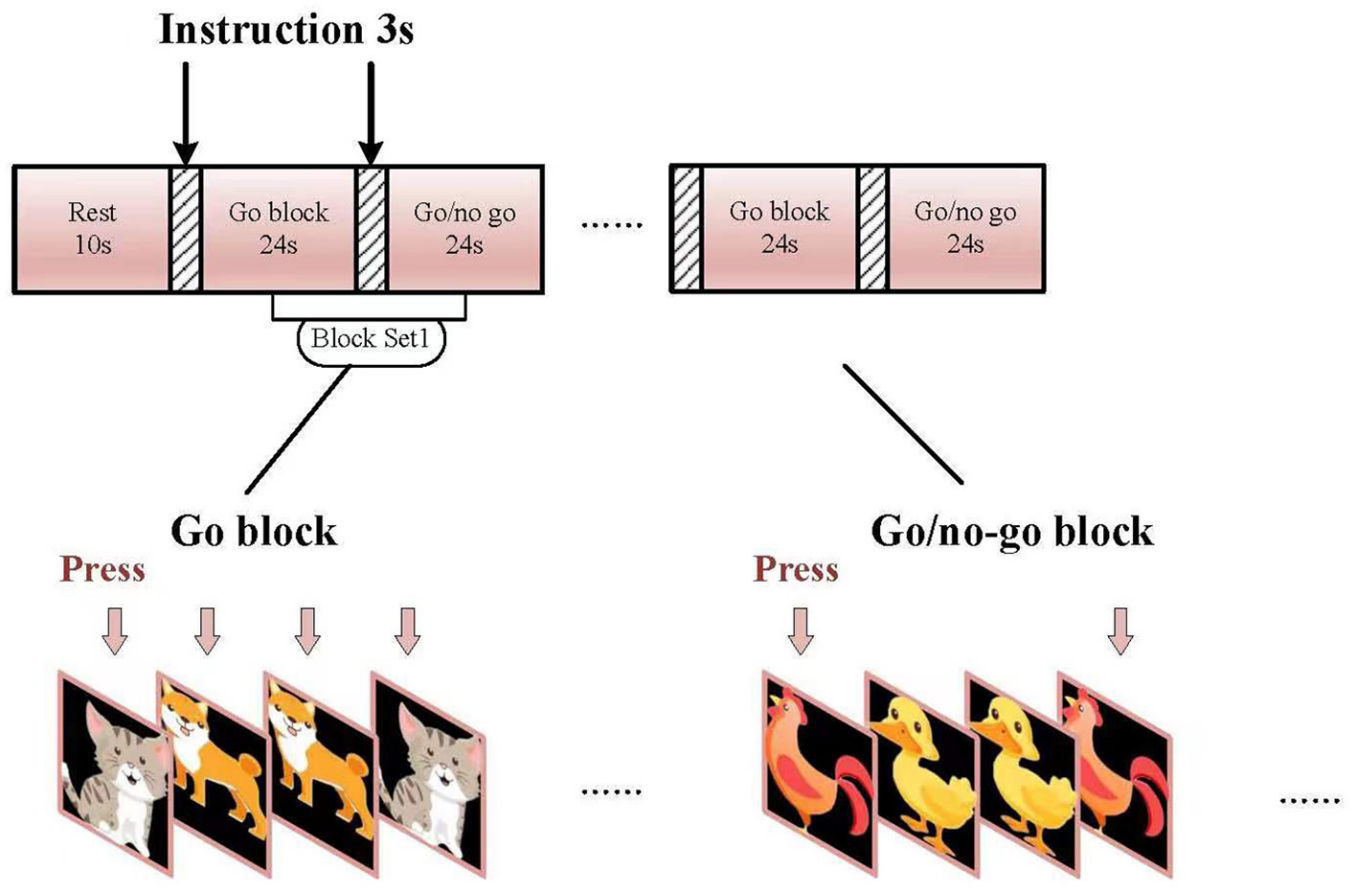
Experimental design.

**Figure 2:**
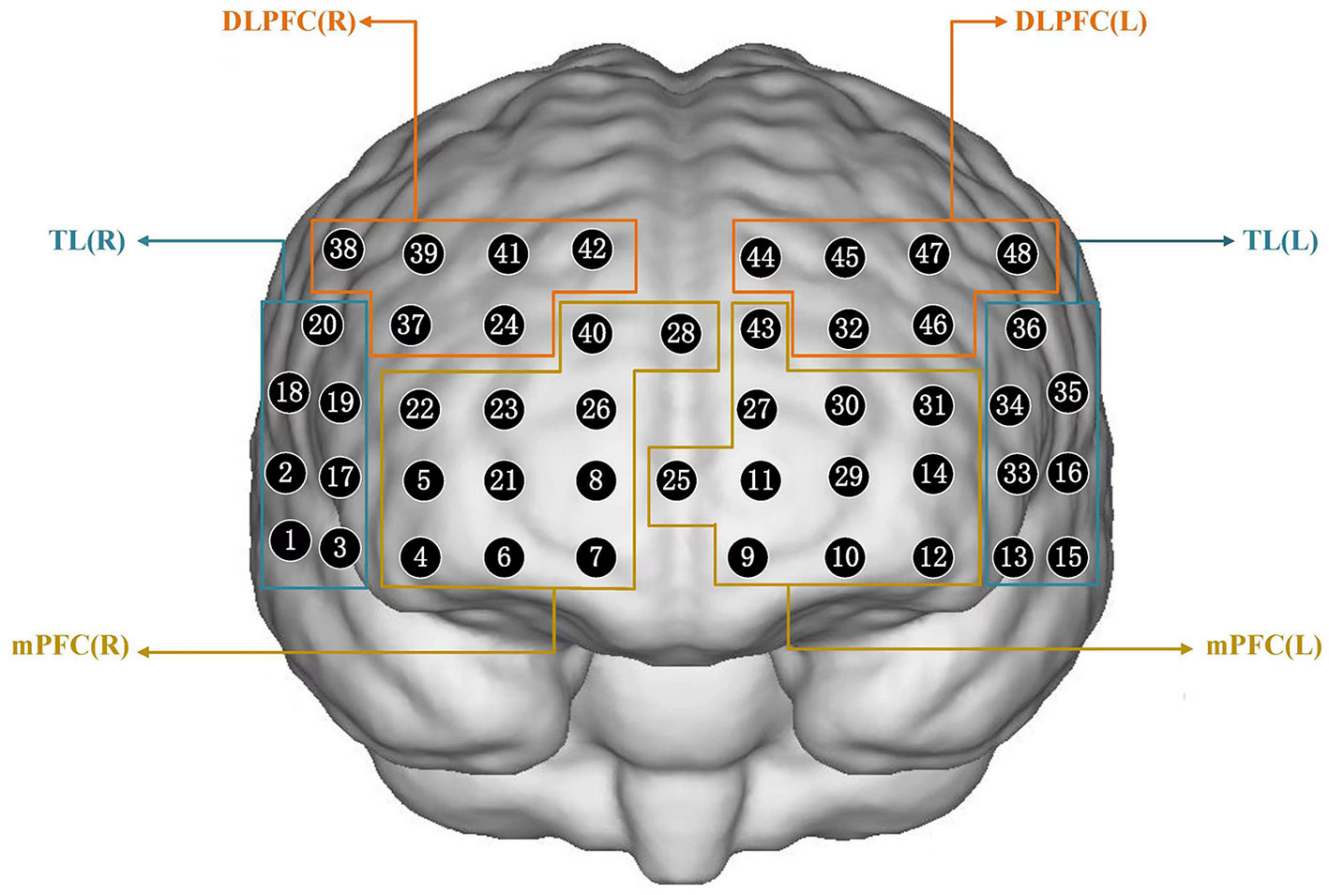
Schematic of arranged fNIRS channels. DLPFC, dorsolateral prefrontal cortex; mPFC, medial prefrontal cortex; TL, temporal lobe; R, right; L, left.

The go/no-go task was organized into 6-min block sets with a 10-s rest. Each set consisted of alternating go (baseline) and go/no-go (target) blocks (Figure 1). Each block contained instructions for 3 s at the beginning of the task, and each condition lasted for 24 s. The total session lasted for 6 min. In the “go” block, participants were displayed with a sequence of two pictures (“cat” and “dog”) and asked to press a key using their right index fingers for both pictures. In the “go/no-go” block, participants were instructed to initiate a response when the picture of a chicken was presented and not initiate a response to the picture of a duck. Each block of task conditions comprised a total of 24 trials. In total, 50% of the trials contained a picture from the “go/no-go” block and were presented in a pseudo-randomized order. Equal numbers of “go” and “go/no-go” pictures were presented to decrease the likelihood of changes in cerebral activity between the two groups as shown earlier (Liddle et al., 2001). All subjects were examined at least once before treatment and were subjected to one practice block before measurements were taken. The experimental design for this experiment is shown in Figures 1, 2. The reaction time (RT) of each trial was recorded to determine accuracy (ACC).

### Data processing and analysis

fNIRS data processing was performed *via* NirSpark analysis software using the following method. (1) Unsatisfactory time intervals containing sudden, obvious, and discontinuous noise were excluded; (2) artifacts induced by motion and the environment were corrected (when the standard deviation of the threshold is 6.0, the amplitude of the threshold is 0.5); (3) a band-pass filter (0.01–0.2 HZ) was applied to remove the slow drift induced by physiological and environmental noise; (4) raw optical density values were converted into concentration changes for both oxygenated hemoglobin (oxy-Hb) and deoxygenated hemoglobin (deoxy-Hb) through the modification of the Beer–Lambert Law; (5) calculation of the inter-trial mean of differences between oxy-Hb concentration changes during the target periods (4–27 s after the go block onset) in each channel were performed for multiple trials (Li et al., 2022); (6) the regional value of difference between changes in oxy-Hb during the target period and baseline was extracted by averaging the categorized channels based on the specified region of interest (ROI); and (7) subjects containing more than three blocks and/or 10 channels were eliminated due to poor signal quality.

Oxy-Hb was selected as the primary indicator for this study due to its higher sensitivity (Strangman et al., 2002; Hoshi, 2003) and better signal-to-noise ratio than deoxy-Hb (Strangman et al., 2002). To better explore the differences in the fNIRS data, the meanΔavg oxy-Hb was calculated for the ROI in each group.

### Behavioral data analysis

To check for behavioral performance differences between the ADHD and TD groups during go/no-go task administration, the reaction time (RT) of go trials and the accuracy (ACC) for go and no-go trials were used as dependent variables. The accuracy for each condition was computed by dividing the correct answer (correct response and appropriate rejection) by the total number of stimuli.

### Statistical analysis

To better compare numerical variables between the ADHD and TD groups, an independent sample *t*-test or chi-square (χ2) test was used to compare data in each category (i.e., clinical characteristics, behavioral performances, and Δavg oxy-Hb). All statistical analyses were conducted using the SPSS statistical software package (version 25.0) with a statistical threshold *p*-value of <0.05.

## Results

### Demographic and clinical characteristics

The baseline demographic characteristics of each study participant are presented in Table 1. ADHD and TD groups contained no difference in their mean ages, gender, or FIQs. As expected, children with ADHD show significantly higher SNAP-IV scores than those in the TD group.

**Table 1:** Demographic and clinical characteristics of participants.

	**ADHD (*****n*** = **32)**		**TD (*****n*** = **31)**			
	**Mean**	**SD**	**Mean**	**SD**	**X** ^2^ **/** ***t***	***p***
**Demographic**						
Age (years)	9.53	1.44	10.10	1.27	−1.65	0.10
Girls: boys	4:28		7:24		1.11	0.29
FSIQ	99.75	4.86	101.0	4.42	1.04	0.30
**SNAP-IV**						
SNAP-IV IA	2.37	1.12	0.75	0.37	7.64	<0.001^***^
SNAP-IV IH	1.24	0.71	0.43	0.35	5.76	<0.001^***^
SNAP-IV ODD	1.73	1.12	0.71	0.50	4.64	<0.001^***^

ADHD, attention-deficit hyperactivity disorder patient group; TD, typically developing peer group; SD, standard deviation; FSIQ, full-scale intelligence quotient; SNAP-IV IA, inattention subscale scores; SNAP-IV IH, hyperactivity subscale scores; SNAP-IV ODD, oppositional defiance.

^***^p < 0.001.

### Behavioral performance

The average accuracy rates and RTs in each go/no-go task for ADHD and TD participants are summarized in Table 2. No significant differences in behavioral performance were observed between the conditions for ADHD and TD participants.

**Table 2:** Go/no-go task performance for ADHD and control groups.

	**ADHD**		**TD**			
	**Mean**	**SD**	**Mean**	**SD**	***t***	***p***
Accuracy-go trail (%)	92.87	6.77	92.28	5.72	−0.375	0.709
Accuracy-no go trail (%)	92.32	7.91	92.09	6.59	−0.125	0.901
RT-go trail (ms)	471.2903	71.40224	489.3750	53.99746	1.136	0.260

RT, reaction time.

### fNIRS results: Δavg oxy-Hb changes

**Figure 3:**
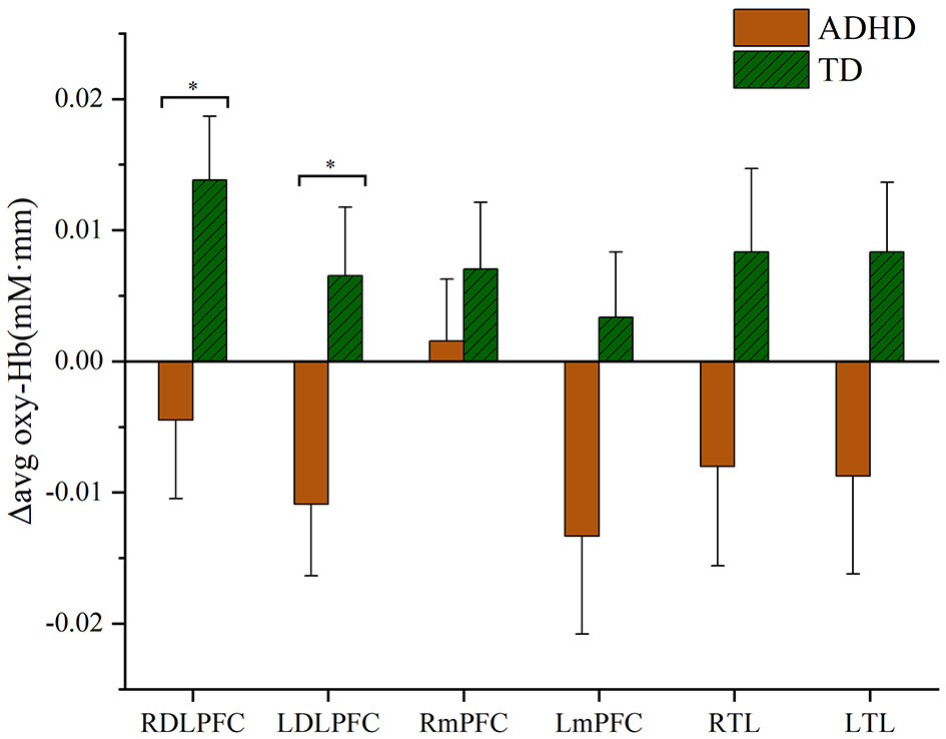
Hemodynamic changes during the performance of the go/no-go task. RDLPFC, right dorsolateral prefrontal cortex; LDLPFC, left dorsolateral prefrontal cortex; RmPFC, right medial prefrontal cortex; LmPFC, left medial prefrontal cortex; RTL, right temporal lobe; LTL, left temporal lobe. *p < 0.05.

Differences in oxy-Hb signals measured using fNIRS are presented according to corresponding areas of the brain and participant group. Compared with the TD group, Δavg oxy-Hb changes in subjects with ADHD were shown to be significantly lower in both right and left DLPFC compared to the TD group (ADHD vs. TD, right DLPFC *t* = −2.364, *p* < 0.05; left DLPFC *t* = −2.301, *p* < 0.05), as shown in Figure 3. Furthermore, this study suggests no significant increase of Δavg oxy-Hb signal in the mPFC in either ADHD or TD groups (ADHD vs. TD, right mPFC *t* = −0.788, *p* = 0.43; left mPFC *t* = 1.849, *p* = 0.069) and TL (right TL *t* = −1.65, *p* = 0.104; left TL *t* = −1.858, *p* = 0.068), as shown in Figure 3.

## Discussion

This study examined the neural substrate of motor response inhibition and monitored performance during a go/no-go task utilizing event-related functional fNIRS. This study investigated the feasibility of using fNIRS in children with ADHD during a go/no-go task administration. In our study, there were no differences in performance between both the ADHD and TD groups, as reported in previous studies (Durston et al., 2003; Smith et al., 2006; Nagashima et al., 2014; Miao et al., 2017). Conversely, we observed that the Δavg oxy-Hb in the right and left DLPFC was significantly lower in the ADHD group compared to the TD group post-administration of the go/no-go task. These results are consistent with several previous studies, which indicated that children with ADHD showed decreased prefrontal activation during go/no-go task administration (Inoue et al., 2012). In addition, a recent fNIRS study by Miao et al. (2017) found that children with ADHD exhibited reduced brain activity in the left FPC during go/no-go task blocks relative to healthy individuals. Furthermore, our results corroborate those of former fMRI findings by Passarotti et al. (2010) and Rubia et al. (2010), which showed a significant reduction in regional bilateral cerebral blood flow to the PFC of children with ADHD during the administration of inhibitory tasks.

Most of the previous fNIRS studies employed near-infrared spectroscopy imaging technology in combination with inhibitory tasks; however, the results of these studies are inconsistent. For example, in 2009, Moser et al. recruited 12 boys with ADHD to take part in a study measuring prefrontal brain activation using fNIRS during the administration of a Stroop task. The results of this study revealed a significant increase in DLPFC activation in children with ADHD compared to healthy controls (Moser et al., 2009). Furthermore, a more recent fNIRS study conducted in 2010 showed that a Stroop task decreased inferior prefrontal cortex activation in children with ADHD compared to controls (Negoro et al., 2010). A recent study by Xiao et al. (2012) explored the impairment of response inhibition using fNIRS in children with ADHD who executed both go/no-go and Stroop tasks. The results of this study revealed that ADHD-affected children contain a lower level of oxyhemoglobin concentration in the PFC during the administration of the go/no-go task compared to the TD group, which is consistent with other studies. However, in this investigation, no significant differences were found between either group with regard to PFC activity during the administration of the Stroop task. More importantly, different cognitive tasks may have an impact on the outcome of changes in brain function. In total, two recent fNIRS studies revealed that subjects suffering from ADHD show higher left DLPFC activity when executing a working memory task (Jang et al., 2021; Calub et al., 2022). Therefore, the brain areas associated with task performance in each study were not consistent, thus rendering the current understanding of the neurobiological basis for attention deficit in patients with ADHD insufficient. Cognition is a complex and multifaceted construct that is hypothesized to involve multiple processes governed by several brain regions (Nowrangi et al., 2014).

Taking all of these studies into account, it can be inferred that brain dysfunction in children with ADHD is associated with functional impairments in the DLPFC. Notably, the go/no-go task is known to activate the DLPFC in a bilateral manner.

Our study confirmed functional near-infrared spectroscopy as a useful measurement tool for studies involving neurodevelopment (Chen et al., 2020), especially with regard to analyzing the effects of interventions for children with ADHD (Grazioli et al., 2019). For example, a recent fNIRS study by Li et al. (2022) examined brain function before and after methylphenidate (MPH) treatment for children with ADHD during the administration of a go/no-go task. These results showed that the average oxygenated hemoglobin concentration as well as expression of the SNAP-25 gene were significantly increased in both the right and left DLPFC of children with ADHD after 4 weeks of MPH treatment. Therefore, fNIRS is a promising imaging tool for the estimation of target interventions. Given that accumulating experimental evidence has pointed to a high-value relationship between cognitive impairment and specific brain regions involved in ADHD, future studies on the neurodevelopment of children and pediatric psychiatry may benefit from functional brain imaging methodologies such as fNIRS.

## Limitations

For data interpretation, our study also presents some strengths. To our knowledge, our study in this field conducted on a completely drug-naive sample a condition necessary to exclude possible drug-related neurobiological effects. Moreover, although we removed some subjects due to their poor fNIRS signal quality, our findings of prefrontal dysfunction in ADHD by fNIRS involved a comparatively large group, allowing for high confidence in the data. However, it is important to address several limitations that our study presents. First, prior studies involved differences in sex, such as hypofrontality, in male subjects only. This study did not perform subgroup analyses by sex due to the limited sample size. Future studies need to replicate these findings independently with larger numbers of patients and according to age and sex. Second, fNIRS only measures Hb concentration changes in upper cortical areas and does not provide measurements at subcortical levels as well as cortical-subcortical connectivity.

## Data availability statement

The original contributions presented in the study are included in the article/Supplementary material, further inquiries can be directed to the corresponding authors.

## Ethics statement

Ethical review and approval was not required for the study on human participants in accordance with the local legislation and institutional requirements. Written informed consent to participate in this study was provided by the participants' legal guardian/next of kin. Written informed consent was obtained from the individual(s), and minor(s)' legal guardian/next of kin, for the publication of any potentially identifiable images or data included in this article.

## Author contributions

TW and XL: experimental design, data collection, data processing, and manuscript writing. FC: data collection. CL and SW: test task writing and data processing. WZ and DZ: experimental design and project implementation management. All authors contributed to the article and approved the submitted version.
