# Supplementary figures and images for: Dorsolateral prefrontal cortex dysfunction caused by a go/no-go task in children with attention-deficit hyperactivity disorder: A functional near-infrared spectroscopy study
Source: Front Neurosci. 2023 Mar 28;17:1145485. doi: 10.3389/fnins.2023.1145485 (PMC10086251; doi:10.3389/fnins.2023.1145485)

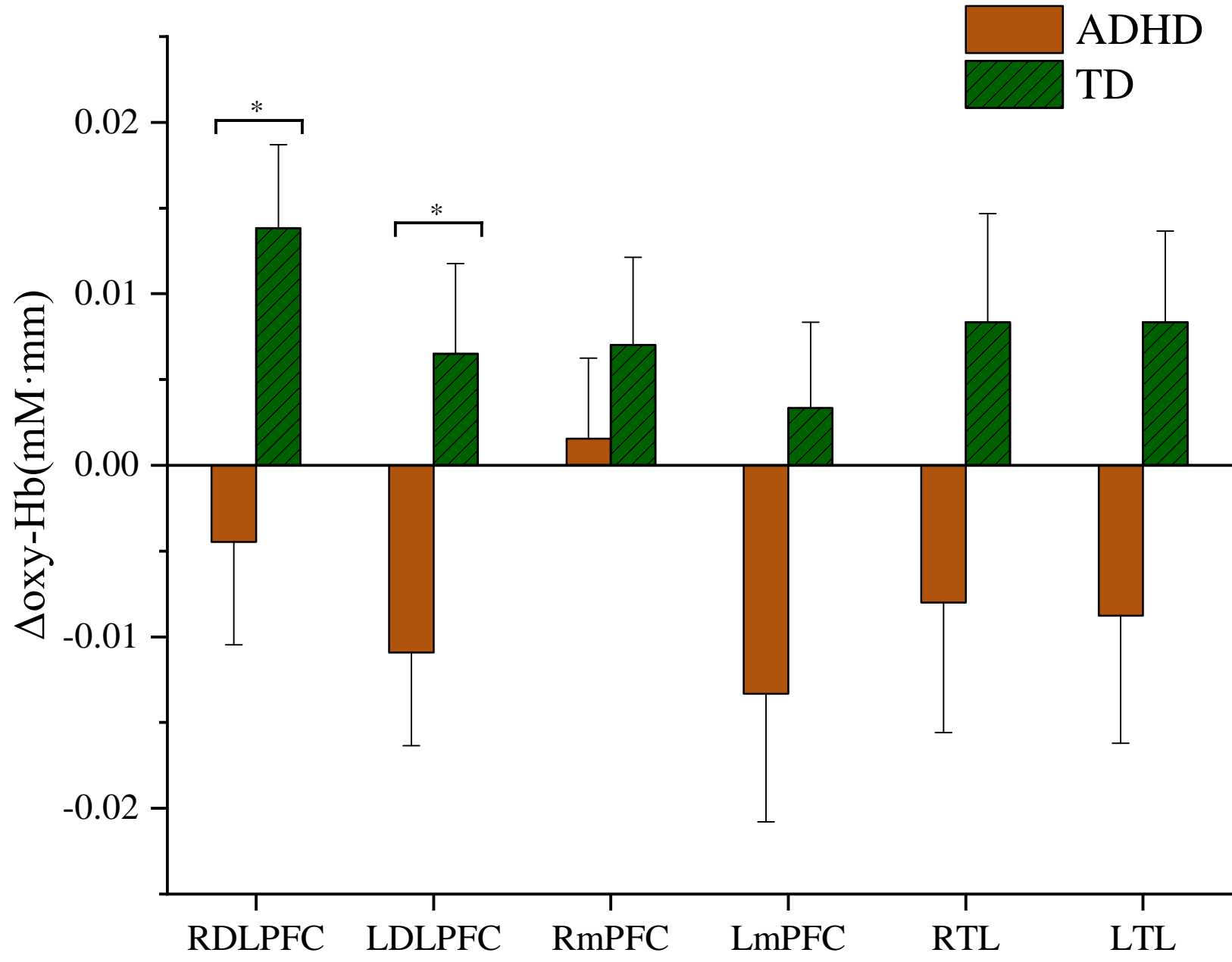

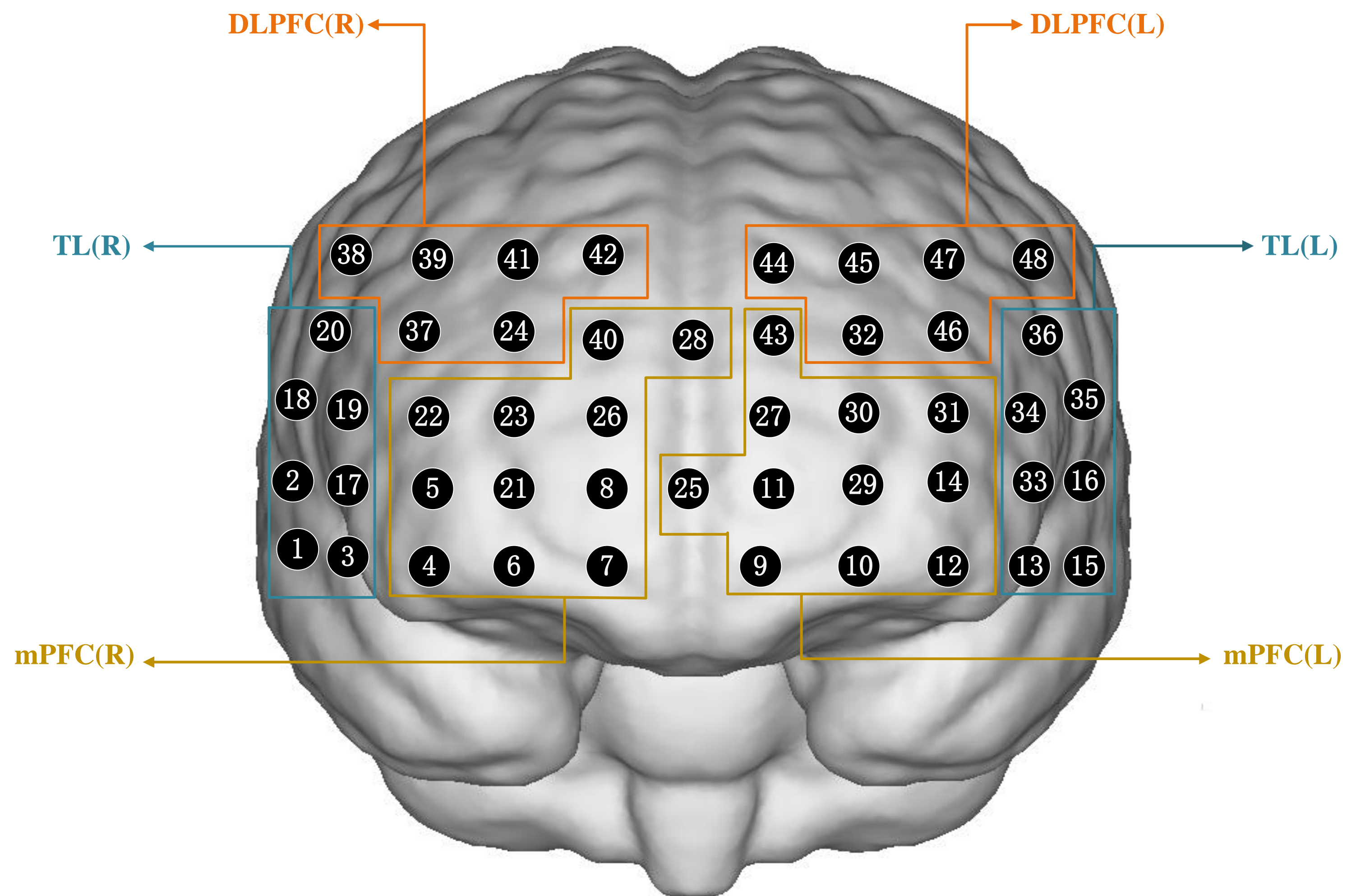

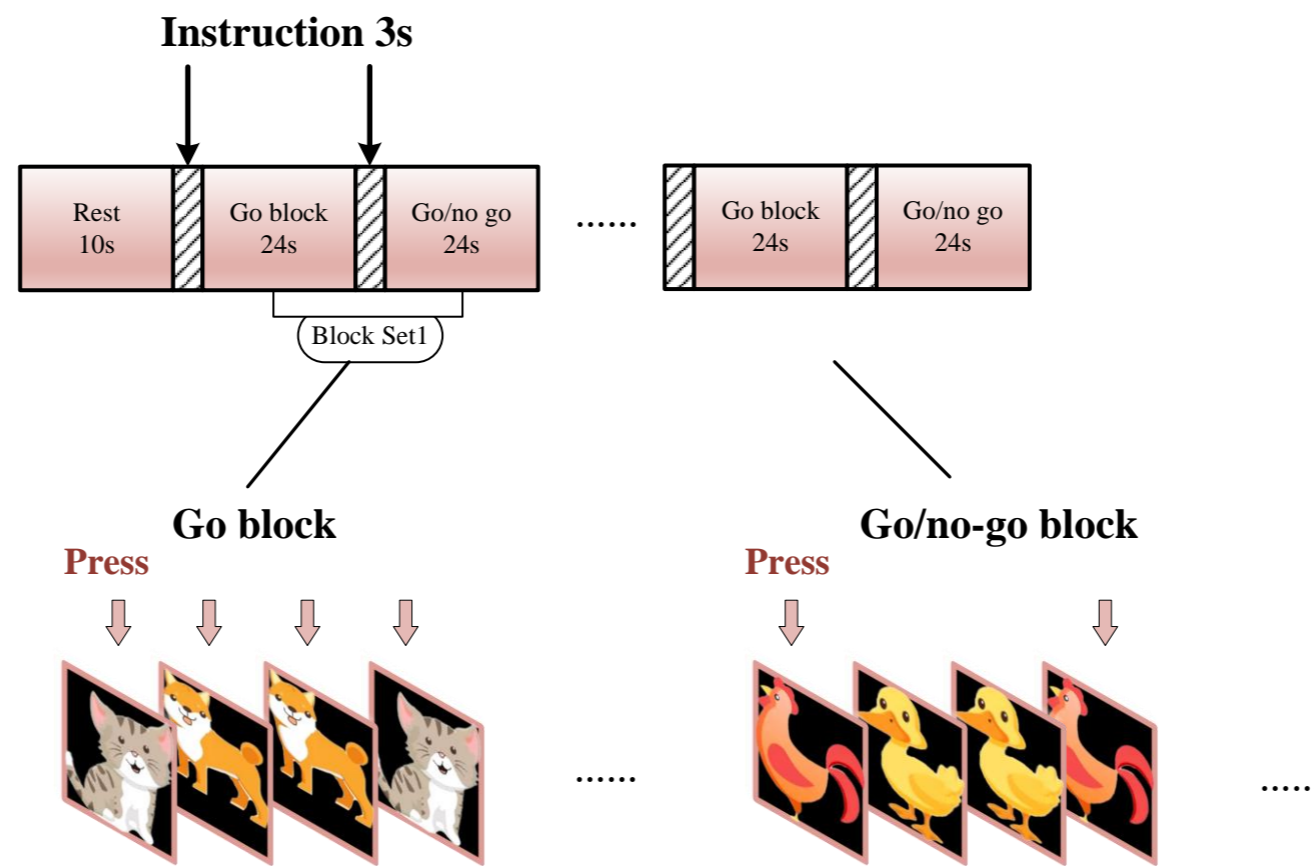

Supplement: Supplementary file 1 [file Image_1.pdf]
